# New Advances in Female Pelvic Floor Dysfunction Management

**DOI:** 10.3390/medicina59061010

**Published:** 2023-05-24

**Authors:** Andrea Braga, Maurizio Serati

**Affiliations:** 1 Department of Obstetrics and Gynecology, EOC—Beata Vergine Hospital, 6850 Mendrisio, Switzerland; 2 Faculty of Biomedical Sciences, Università della Svizzera Italiana, 6900 Lugano, Switzerland; 3 Department of Obstetrics and Gynecology, Del Ponte Hospital, University of Insubria, 21100 Varese, Italy

Pelvic floor dysfunctions (PFDs), which include various disorders such as urinary and anal incontinence, pelvic organ prolapse, and sexual disorders, are widespread amongst females, so much so that they affect one fifth of adult women. These disorders represent serious physical and psychological distress, with a great impact on quality of life and self-esteem, leading to a depressed status. In recent years, an increasing number of women have reported the need for treatment for these issues, with an enormous economic burden. In addition, considering the general aging of the world population, the demand of care will further increase in the future. Recently, new advances in the management of female PFD have been proposed. This Special Issue of *Medicina*, entitled “New advances in Female Pelvic Floor Dysfunctions Management”, includes nine articles dealing with the recent advances in the diagnosis and treatment of PFDs. Most of the studies have focused their research on the management of urinary incontinence (UI), especially stress urinary incontinence (SUI), which is known to be the most common type of UI [1].

In the well-conducted review by Yang et al. [2], the authors analyzed the pathogenesis of SUI from three different perspectives, including the anatomical factors of the urethra itself, the periurethral area, and the pelvic floor nerve. Among the many anatomical factors considered, they believe that the joint contraction of the levator ani muscle and the external urethral sphincter, leading to the formation of the urethral bend angle and to the urethra being forcefully closed, plays a key role in UI. Therefore, the treatment of SUI should focus on the repair and reconstruction of these structures on the basis of the current theory of mid-urethral suspension. Secondly, they emphasize that the sling surgical approach to SUI needs to be improved based on the anatomical pathogenesis.

Although the role of mid-urethral slings (MUSs) has recently been questioned due to reports of vaginal mesh complications, they remain the gold standard for SUI treatment [3]. However, more information regarding the long-term outcomes and mesh complications is needed to confirm this role. Braga et al. [4], in a prospective study on fifty women with urodynamic SUI, evaluated the efficacy and safety of the tension-free vaginal tape abbrevo (TVT-A) procedure at medium-term follow-up. Five years after TVT-A implantation, 84.4% and 88.9% of patients were subjectively and objectively cured, respectively, without serious complications or groin–thigh pain. A significant trend of de novo overactive bladder (OAB) symptoms was found, as reported by 22.2% at the last evaluation. The rate of onset of de novo OAB does not differ to the traditional MUSs [1,5] and could reflect the aging of the patients rather than being a direct consequence of surgery. In conclusion, the authors stated that TVT-A seems to be an effective option for the treatment of SUI, with very low complication and groin–thigh pain rates. Furthermore, as reported by Tian et al. [6], the trans-obturator approach seems to also be effective when combined with pelvic floor reconstructive surgery.

Other new non-invasive therapies seem to play a role in the treatment of UI, such as low-intensity extracorporeal shock wave therapy (LIESWT) [7]. The extracorporeal shock wave is a longitudinal acoustic wave that propagates through human tissues at the speed of an ultrasound wave in water, promoting angiogenesis, recruiting mesenchymal stem cells and endothelial progenitor cells, stimulating cellular proliferation and regeneration, and inhibiting oxidative stress, thus improving blood circulation and enhancing tissue repair. Lin et al. [7] in a multicenter, single-blind, randomized controlled trial study, on sixty female SUI patients, demonstrated that 8 weeks of LiESWT could improve SUI symptoms during physical activity, reduce urine leakage, lessen OAB symptoms, and promote QoL. However, future research will have to confirm these preliminary results.

Pulsed magnetic stimulation is another non-surgical approach that has become popular in the last few years, especially for the treatment of SUI. The device has been approved by the Food and Drug Administration (FDA) as a conservative treatment for UI since 1998, and it has already shown effective results in previous studies [8,9]. It generates electrical activity, which induces controlled depolarization of the nerves, resulting in pelvic muscle contraction and sacral S2–S4 root neuromodulation [9]. In this Special Issue, González-Isaza et al. [8], in a case–control study, showed that pulsed magnetic stimulation is a safe and attractive non-invasive alternative for patients who prefer non-surgical treatments, although the data are available until 14 weeks of follow-up.

In addition, the effects of non-invasive radiofrequency diathermy (RFD) on PFD were evaluated by González-Gutiérrez [10]. This systematic review included 15 studies and showed improvements in UI, pelvic pain conditions, pelvic floor muscle strength, and sexual function with the use of RFD, although the quality of most of these studies was low. In fact, the authors stated that these findings must be considered with caution until more randomized clinical trials are performed to solve the biases detected.

The last but not least topic covered in this Special Issue by Frigerio et al. [11] surrounds the urodynamic (UDS) evaluation of patients with UI. UDS is considered the gold standard for lower urinary tract functional assessment; however, it requires very specific skills and training, which are currently difficult to master due to its limited use. The authors in this prospective study showed a resident without previous experience how to correctly perform and interpret UDS and reach technical autonomy in the execution of uroflowmetry and cystomanometry with a pressure/flow study, after 6–10 and 16–20 procedures, respectively.

They found that there is a tangible learning curve for UDS in terms of several proficiency parameters.

Overall, the papers published in this Special Issue contribute to a deeper understanding of the pathophysiology of PFD, addressing the appropriate therapies to reduce the effects of these disorders on women’s quality of life. This should be a priority for future research.

## Data Availability

Not applicable.
